# *In vitro* follicle growth supports human oocyte meiotic maturation

**DOI:** 10.1038/srep17323

**Published:** 2015-11-27

**Authors:** Shuo Xiao, Jiyang Zhang, Megan M. Romero, Kristin N. Smith, Lonnie D. Shea, Teresa K. Woodruff

**Affiliations:** 1 Department of Obstetrics and Gynecology, Feinberg School of Medicine, Northwestern University, Chicago, IL 60611, USA; 2 Center for Reproductive Science, Northwestern University, Evanston, IL 60208, USA; 3 Master of Biotechnology Program, Northwestern University, Evanston, IL 60208, USA; 4 Northwestern Medical Group, Northwestern University, Chicago, IL, 60611, USA; 5 Department of Biomedical Engineering, College of Engineering and Medical School, University of Michigan, Ann Arbor, MI 48109, USA

## Abstract

*In vitro* follicle growth is a potential approach to preserve fertility for young women who are facing a risk of premature ovarian failure (POF) caused by radiation or chemotherapy. Our two-step follicle culture strategy recapitulated the dynamic human follicle growth environment *in vitro*. Follicles developed from the preantral to antral stage, and, for the first time, produced meiotically competent metaphase II (MII) oocytes after *in vitro* maturation (IVM).

With significantly improved survival rates for young women and girls diagnosed with cancer and other life-threatening diseases, there is increased awareness of the risk of premature ovarian failure (POF) from radiation or chemotherapy and the possibility of preserving fertility prior to starting treatment^1^,^2^,^3^,^4^. The most successful fertility preservation options, oocyte and embryo cryopreservation, require a delay in cancer treatment for hormone stimulation to retrieve mature oocytes, which may not be an option for patients with aggressive cancers. Further, hormone stimulation is not possible for prepubertal girls. Another option is ovarian tissue cryopreservation; however, tissue transplantation carries a risk of reintroducing cancer cells into the patient^1^.

*In vitro* follicle growth has great potential to provide an additional fertility preservation option for young women and girls with cancer^5^. This technique does not require hormone stimulation, is available to both reproductive-age women and prepubertal girls, and could avoid the risk of re-exposure of cancer cells in transplantation^1^. Several *in vitro* follicle culture systems have successfully supported the growth and maturation of ovarian follicles in mice and several large mammalian species^6^. However, translation of this work to humans has been challenging, and to date, *in vitro* growth and development of meiotically competent human oocytes from preantral follicles has not been achieved^7^,^8^. The objective of this study was to develop a two-step follicle culture strategy to recapitulate the dynamic human follicle growth environment and support follicle and oocyte maturation *in vitro*.

## Results and Discussion

**Figure 1:**
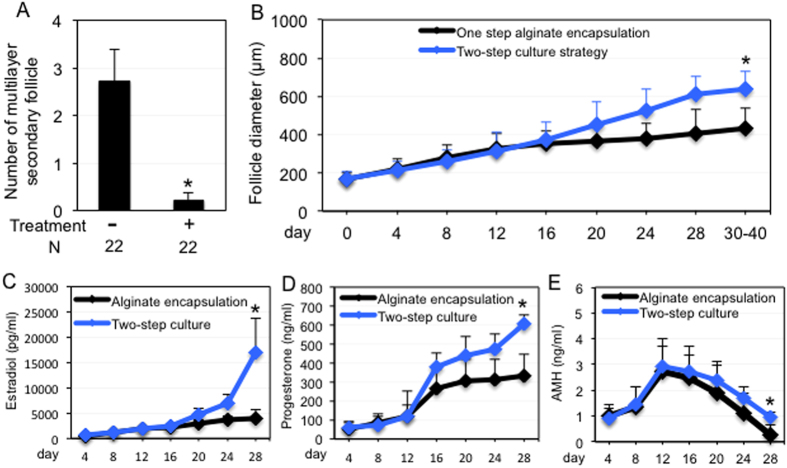
The two-step follicle culture strategy promoted follicle growth and hormone production. (**A**) Average number of follicles collected from patients with or without previous cancer treatments. (**B**) Growth of follicles cultured only within alginate hydrogels or using the two-step culture strategy. (**C–E**) Hormone secretion from growing follicles cultured in alginate hydrogels or using the two-step strategy. (**C**) Estradiol, (**D**) Progesterone, and (**E**) AMH. *p < 0.05 compared to follicles culture with alginate hydrogels only.

Human ovarian tissues were obtained from 44 patients from the National Physician Cooperative (NPC) of the Oncofertility Consortium (oncofertility.northwestern.edu), with 72.7% of the patients were younger than 20 years of age, 20.5% of the patients were between 20 and 30 years of age, and 6.8% of the patients were older than 30 years of age (Table S1). All patients had a cancer diagnosis, and 22 patients had undergone radiation and/or chemotherapy prior to ovarian tissue removal (Table S1). We mechanically isolated 65 multilayer secondary follicles (Fig. S1A,B), with significantly fewer follicles collected from tissues from women who had previous cancer treatment (Fig. 1A), thus confirming the gonadotoxic effects of cancer treatments^3^.

Culture of human follicles on a flat surface irreversibly disrupted the interaction between the oocyte and its surrounding granulosa cells (Fig. S1C), and oocytes underwent degeneration within 4 to 6 days of culture (Fig. S1D). In our follicle culture system, an alginate hydrogel-based encapsulation method is used to maintain the three-dimensional follicle architecture^9^; this is the only follicle culture technology that has successfully resulted in meiotically competent oocytes and cleavage-stage embryos in the non-human primate^10^. Thus, human follicles were encapsulated and cultured in 0.5% alginate hydrogels with initial diameter of follicle and oocyte at 165.8 ± 32.3 μm and 73.5 ± 2.1 μm, respectively. By 10–15 days of culture, 32 follicles started to display an antrum formation and had a diameter of 400–500 μm (Fig. S2).

Our group previously reported that human follicles cultured within alginate hydrogels produced oocytes with terminal diameters of 110 μm on day 30^7^; however, none of these oocytes reached metaphase II (MII) stage after *in vitro* maturation (IVM). In mouse follicle culture, decreasing the concentration of alginate significantly improved mouse oocyte maturation^11^. In humans, immature follicles gradually move from the rigid collagen-dense cortex zone to the less dense perimedullary region as they grow^12^,^13^,^14^. Therefore, to mimic *in vitro* the shift from a rigid cortex to permissive perimedullary region observed *in vivo*, we released a portion of follicles from the alginate hydrogels at the antral stage, and then continued the cultures in low-attachment plates for up to 40 days. Compared to follicles cultured in the alginate hydrogels, follicles cultured using this two-step strategy had significantly greater terminal diameters starting on day 20 (Fig. 1B), suggesting that follicles require a stage-specific growing environment.

We next examined the hormone expression patterns of cultured follicles. Both estradiol and progesterone levels were positively correlated to follicle development^15^ (Fig. 1C,D), and anti-Müllerian hormone (AMH) transiently increased during the early stages of follicle growth and then declined upon antrum formation (Fig. 1E). After release from alginate encapsulation, follicles had significantly higher hormone levels, indicating that the two-step culture strategy permits an increase in follicle hormone production during later stages of follicle development.

**Figure 2:**
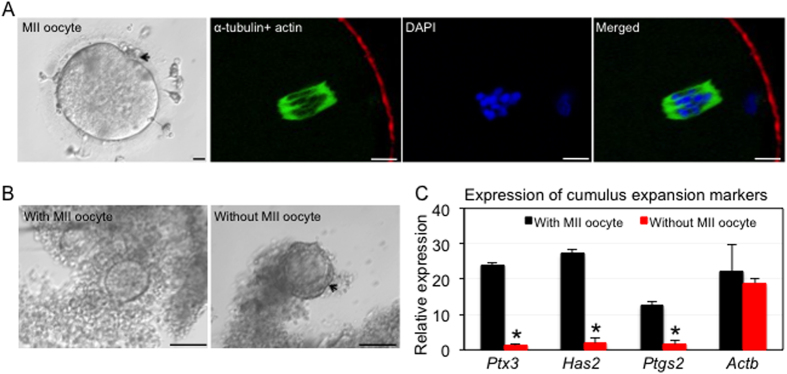
The two-step follicle culture strategy supported human oocyte meiotic maturation. (**A**) Representative images of an MII oocyte with the first polar body extrusion after IVM, and its corresponding meiotic spindle (green), actin (red), and chromosomes (blue) after immunofluorescence staining. (**B**) Cumulus expansion of oocytes that did or did not reach MII. (**C**) Expression of cumulus expansion markers after IVM. The housekeeping gene *Gapdh* (Glyceraldehyde 3-phosphate dehydrogenase) was used as a loading control and *Actb* (Beta-actin) was used as a positive control. *p < 0.05 compared to cumulus cells with oocytes reach MII; black arrow: (**A**) polar body of MII oocyte and (**B**) cumulus cells tightly associated to the egg that did not reach MII; scale bar: 10 μm in A and 100 μm in B.

IVM was performed to determine whether the two-step follicle culture strategy promotes oocyte maturation. Follicles cultured only with alginate encapsulation produced oocytes that either remained in the germinal vesicle (GV) stage (8 out of 12) or degenerated (4 out of 12) (Fig. S3A-D). In contrast, 20% (4 out of 20) of follicles cultured using the two-step strategy produced meiotic competent MII oocytes that extruded the first polar body, and with barrel-shaped bipolar spindles that were perpendicular to the oocyte membrane and with tightly aligned chromosomes on the metaphase plate (Fig. 2A, and Table S2). The oocytes that reached the MII stage had diameters of 111.4 ± 2.1 μm, which was not significantly different from the oocytes that remained in GV stage (110.62 ± 1.73). Our results demonstrate that the oocyte meiotic competence could be achieved although the terminal follicle size couldn’t reach the size of preovulatory follicle size (15–20 mm) *in vivo*. This is consistent with a previous report that culturing human preantral follicles to the preovulatory diameter is possibly unnecessary as both macaque and human oocyte retrieved from smaller antral follicles produced blastocyst stage embryos and live birth, respectively^16^,^17^,^18^.

Human follicles cultured in alginate for 30 days produced GV stage oocytes with tightly compacted cumulus cells; while those follicles that reached MII (two-step cultures) had cumulus cells that responded to hormone stimulation with cumulus expansion after IVM (Fig. 2B), and with significantly higher expression levels of pentraxin-related gene (*Ptx3*), hyaluronan synthase 2 (*Has2*), and prostaglandin-endoperoxide synthase 2 (*Ptgs2*) (Fig. 2C), which are critical for oocyte meiosis and the subsequent developmental competence^19^,^20^. Current federal law restricts our ability to test the human oocyte developmental competence through parthenogenesis or *in vitro* fertilization (IVF)^21^; therefore, we demonstrated the oocyte developmental competence by positive expression of proteins known to be critical for oocyte development, TPX2 (targeting protein for the *Xenopus* kinesin xklp2) and DAZL (deleted in azoospermia-like) (Fig. S3E,F), which colocalize with the oocyte spindle and distribute throughout the oocyte cytoplasm, respectively^22^,^23^,^24^.

The human follicle is difficult to culture *in vitro* because it requires extended culture time periods and reaches a larger terminal size compared to the follicles of other species. Our study demonstrates that human follicle growth and oocyte maturation *in vitro* requires a dynamic environment. By using a two-step culture strategy, follicles could be grown from the preantral to antral stage, and, for the first time, produced meiotically competent MII oocytes. Continued advances in follicle culture protocols to support the production of high-quality, meiotically and developmentally competent oocytes will one day provide an additional fertility preservation option for young women and girls facing diseases or treatments that threaten their reproductive health.

## Methods

### Human ovarian tissue collection, follicle isolation, encapsulation and culture

Human ovarian tissues were obtained from participants following informed consent under an Institutional Review Board (IRB) approved protocol at Northwestern University. All experiments, procedures, and methods were carried out in accordance with the IRB approved guidelines and regulations. Under this protocol, participants consent to donate 20% of their ovarian tissue for research on developing *in vitro* human follicle growth technology through the National Physicians Cooperative (NPC) of the Oncofertility Consortium (oncofertility.northwestern.edu). Ovarian cortical strips were cut into 1 mm^3^ pieces in the dissection media (Leibovitz L-15, Invitrogen, Carlsbad, CA, USA) supplemented with 1% fetal bovine serum (FBS, Life Technology, Grand Island, NY, USA). Follicles were mechanically isolated using 25-gauge needles in the dissection media.

Collected follicles were encapsulated individually in 0.5% alginate (NovaMatrix, Sandvika, Norway). Alginate beads were placed in 96-well plates, with each well containing 100 μl growth media (50% αMEM Glutamax and 50% F-12 Glutamax supplemented with 3 mg/ml human serum albumin [HSA] [Sigma-Aldrich, St. Louis, MO, USA], 10 mIU/ml recombinant follicle-stimulating hormone [rFSH; from A. F. Parlow, National Hormone and Peptide Program, National Institute of Diabetes and Digestive and Kidney Diseases, Bethesda, MD, USA], 1 mg/ml bovine fetuin [Sigma-Aldrich, St. Louis, MO, USA], 5 μg/ml insulin, 5 μg/ml transferrin, and 5 μg/ml selenium [Sigma-Aldrich, St. Louis, MO, USA]). For all experiments, follicles were maintained at 37 °C. Half of the growth media (50 μl) was replaced every other day. Follicles were imaged at each media change. Follicles were considered dead if they had unhealthy appearing oocytes and/or granulosa cells, or if the integrity of the oocyte and somatic cell interface was visibly compromised. Once follicle diameter reached 400–500 μm with antrum formation, a portion of follicles were released from alginate hydrogels and cultured in the low attachment plates (Corning Inc, Corning, NY, USA) for up to 30–40 days.

### Hormone measurements

17β-estradiol (E2) and progesterone (P4), and anti-Müllerian hormone (AMH) concentrations in the follicle culture media were measured using ELISA kits (Calbiotech, Spring Valley, CA, USA for E2 and P4, and AnshLabs, Webster, TX, USA for AMH) according to the manufacturer’s instructions. All assays were run in duplicate and medium collected from wells without follicles was used as negative control.

### *In vitro* maturation (IVM)

IVM, which is the technique of triggering oocyte maturation *in vitro*, was performed after follicles were cultured up to 30–40 days. Follicles were incubated for 16 h at 37 °C in 5% CO_2_ in air in maturation media (αMEM with 10% fetal bovine serum, 1.5 IU/ml human chorionic gonadotropin (hCG), 10 ng/ml epidermal growth factor (EGF) [BD Biosciences, Franklin Lakes, NJ, USA], and 10 mIU/ml rFSH). Oocytes were then denuded from the surrounding cumulus cells using 0.3% hyaluronidase (Sigma-Aldrich, St. Louis, MO, USA). Oocytes with an intact nucleus were considered to be arrested at prophase I in the germinal vesicle (GV) stage. If a polar body was present in the perivitelline space, the oocytes were classified as metaphase II (MII). Fragmented or shrunken oocytes were classified as degenerated (D). Follicle somatic cells were snap frozen in dry ice for quantitative reverse transcription PCR (RT-qPCR).

### Immunofluorescence

Oocytes were fixed in 3.8% paraformaldehyde containing 0.1% Triton X-100 (Sigma-Aldrich, St. Louis, MO, USA) for 1 h at 37 °C, and washed 3 times in blocking solution with 1x PBS containing 0.3% BSA and 0.01% Tween-20. Oocytes were incubated overnight with rabbit anti-α-tubulin (Cell Signaling Technology, Danvers, MA, USA), rabbit anti-TPX2 (Novus, Littleton, CO, USA), and rabbit anti-DAZL (Abcam, Cambridge, MA, USA) in blocking solution. Then, oocytes were washed 3 times with blocking solution and incubated with anti-rabbit IgG for 1 h at RT, mounted using Vectashield containing DAPI (Vector Laboratories, Burlingame, CA, USA), and imaged by confocal (Leica Microsystem, Buffalo Grove, IL, USA).

### Gene Analysis

RT-qPCR was used to determine the expression levels of cumulus expansion markers of pentraxin-related gene (*Ptx3*), hyaluronan synthase 2 (*Has2*), and Prostaglandin-endoperoxide synthase 2 (*Ptgs2*). The cumulus cells were from three follicles with MII oocyte and five follicles with GV oocyte. Total RNA of follicle somatic cells after IVM was isolated using Trizol. cDNA was reverse-transcribed from one microgram of total RNA using Superscript III reverse transcriptase with random primers (Invitrogen, Carlsbad, CA, USA). RT-qPCR was performed in 384-well plates using SYBR-Green I intercalating dye on ABI 7900 (Applied Biosystems, Carlsbad, CA, USA). Primer sequences were *Ptx3*: ACCAATGAGGCTTGAGTCTT (forward) and CTCCCAGAGAAGGCTAATGT (reversed); *Has2*: GTAACGCAATTGGTCTTGTC (forward) and ACCAATCTTCCACAAACTCA (reversed); and *Ptgs2*: TCTGATGATGTATGCCACAA (forward) and CAACAAACTGGGTAATTCCA (reversed); *Gapdh*: GAGATCCCTCCAAAATCAAG (forward) and CTGATGATCTTAGGCTGTT (reversed). The RT-qPCR was repeated three times with the same RNA and cDNA as the limited number of cumulus cell samples.

### Statistical analyses

The average number of follicles collected from patients with and without previous cancer treatment, were compared by two-tail unequal variance Student’s t-test. Follicle diameter, hormone secretion, and gene expression data were analyzed using one-way ANOVA, followed by Tukey range test for significant difference. The significance level was set at *p* < 0.05.

## Additional Information

**How to cite this article**: Xiao, S. *et al. In vitro* follicle growth supports human oocyte meiotic maturation. *Sci. Rep.* **5**, 17323; doi: 10.1038/srep17323 (2015).

## Supplementary Material

Supplementary Information

## References

[b1] Jeruss J. S. & Woodruff T. K. Preservation of fertility in patients with cancer. The New England journal of medicine 360, 902–911, doi: 10.1056/NEJMra0801454 (2009). 19246362 PMC2927217

[b2] Anderson R. A. *et al.* Do doctors discuss fertility issues before they treat young patients with cancer? Hum Reprod 23, 2246–2251, doi: 10.1093/Humrep/Den252 (2008). 18614615

[b3] Morgan S., Anderson R. A., Gourley C., Wallace W. H. & Spears N. How do chemotherapeutic agents damage the ovary? Hum Reprod Update 18, 525–535, doi: Doi 10.1093/Humupd/Dms022 (2012). 22647504

[b4] Anderson R. A. *et al.* Cancer treatment and gonadal function: experimental and established strategies for fertility preservation in children and young adults. The lancet. Diabetes & endocrinology, doi: 10.1016/S2213-8587(15)00039-X (2015). 25873571

[b5] Shea L. D., Woodruff T. K. & Shikanov A. Bioengineering the ovarian follicle microenvironment. Annual review of biomedical engineering 16, 29–52, doi: 10.1146/annurev-bioeng-071813-105131 (2014). PMC4231138 24849592

[b6] De Vos M., Smitz J. & Woodruff T. K. Fertility preservation in women with cancer. Lancet 384, 1302–1310, doi: 10.1016/S0140-6736(14)60834-5 (2014). 25283571 PMC4270060

[b7] Xu M. *et al.* *In vitro* grown human ovarian follicles from cancer patients support oocyte growth. Hum Reprod 24, 2531–2540, doi: 10.1093/humrep/dep228 (2009). 19597190 PMC2743446

[b8] Telfer E. E., McLaughlin M., Ding C. & Thong K. J. A two-step serum-free culture system supports development of human oocytes from primordial follicles in the presence of activin. Hum Reprod 23, 1151–1158, doi: 10.1093/humrep/den070 (2008). 18326514

[b9] Xu M., Kreeger P. K., Shea L. D. & Woodruff T. K. Tissue-engineered follicles produce live, fertile offspring. Tissue engineering 12, 2739–2746, doi: 10.1089/ten.2006.12.2739 (2006). 17518643 PMC2648391

[b10] Xu J. *et al.* Secondary follicle growth and oocyte maturation during encapsulated three-dimensional culture in rhesus monkeys: effects of gonadotrophins, oxygen and fetuin. Hum Reprod 26, 1061–1072, doi: 10.1093/humrep/der049 (2011). 21362681 PMC3079470

[b11] Xu M., West E., Shea L. D. & Woodruff T. K. Identification of a stage-specific permissive *in vitro* culture environment for follicle growth and oocyte development. Biology of reproduction 75, 916–923, doi: 10.1095/biolreprod.106.054833 (2006). 16957022

[b12] Gougeon A. Dynamics of follicular growth in the human: a model from preliminary results. Hum Reprod 1, 81–87 (1986). 3558758 10.1093/oxfordjournals.humrep.a136365

[b13] Tingen C., Kim A. & Woodruff T. K. The primordial pool of follicles and nest breakdown in mammalian ovaries. Molecular human reproduction 15, 795–803, doi: 10.1093/molehr/gap073 (2009). 19710243 PMC2776475

[b14] Woodruff T. K. & Shea L. D. A new hypothesis regarding ovarian follicle development: ovarian rigidity as a regulator of selection and health. J Assist Reprod Gen 28, 3–6, doi: Doi 10.1007/S10815-010-9478-4 (2011). PMC3045494 20872066

[b15] Xu J. *et al.* Survival, growth, and maturation of secondary follicles from prepubertal, young, and older adult rhesus monkeys during encapsulated three-dimensional culture: effects of gonadotropins and insulin. Reproduction 140, 685–697, doi: 10.1530/REP-10-0284 (2010). 20729335 PMC3351200

[b16] Telfer E. E. & Zelinski M. B. Ovarian follicle culture: advances and challenges for human and nonhuman primates. Fertility and sterility 99, 1523–1533, doi: 10.1016/j.fertnstert.2013.03.043 (2013). 23635350 PMC3929501

[b17] Guzman L. *et al.* Developmental capacity of *in vitro*-matured human oocytes retrieved from polycystic ovary syndrome ovaries containing no follicles larger than 6 mm. Fertility and sterility 98, 503–507, doi: 10.1016/j.fertnstert.2012.01.114 (2012). 22365339

[b18] Peluffo M. C., Barrett S. L., Stouffer R. L., Hennebold J. D. & Zelinski M. B. Cumulus-oocyte complexes from small antral follicles during the early follicular phase of menstrual cycles in rhesus monkeys yield oocytes that reinitiate meiosis and fertilize *in vitro*. Biology of reproduction 83, 525–532, doi: 10.1095/biolreprod.110.084418 (2010). 20519694 PMC2957158

[b19] Salustri A. *et al.* PTX3 plays a key role in the organization of the cumulus oophorus extracellular matrix and in *in vivo* fertilization. Development 131, 1577–1586, doi: 10.1242/dev.01056 (2004). 14998931

[b20] McKenzie L. J. *et al.* Human cumulus granulosa cell gene expression: a predictor of fertilization and embryo selection in women undergoing IVF. Hum Reprod 19, 2869–2874, doi: 10.1093/humrep/deh535 (2004). 15471935

[b21] Tingen C., Rodriguez S., Campo-Engelstein L. & Woodruff T. K. Research funding. Politics and parthenotes. Science 330, 453, doi: 10.1126/science.1196881 (2010). 20966235

[b22] Chen J. *et al.* Somatic cells regulate maternal mRNA translation and developmental competence of mouse oocytes. Nature cell biology 15, 1415–1423, doi: 10.1038/ncb2873 (2013). 24270888 PMC4066669

[b23] Cauffman G., Van de Velde H., Liebaers I. & Van Steirteghem A. DAZL expression in human oocytes, preimplantation embryos and embryonic stem cells. Molecular human reproduction 11, 405–411, doi: 10.1093/molehr/gah167 (2005). 15879466

[b24] Chen J. *et al.* Genome-wide analysis of translation reveals a critical role for deleted in azoospermia-like (Dazl) at the oocyte-to-zygote transition. Gene Dev 25, 755–766, doi: 10.1101/gad.2028911 (2011). 21460039 PMC3070937

