# Supplementary material for: In vitro follicle growth supports human oocyte meiotic maturation
Source: Sci Rep. 2015 Nov 27;5:17323. doi: 10.1038/srep17323 (PMC4661442; doi:10.1038/srep17323)
Supplement: Supplementary Information [file srep17323-s1.pdf]

***In vitro* follicle growth supports human oocyte meiotic maturation**

Shuo Xiao[1, 2], Jiyang Zhang[1, 3], Megan M. Romero[1, 2], Kristin N. Smith[4], Lonnie D. Shea[5], and Teresa K. Woodruff [1, 2*]

1. Department of Obstetrics and Gynecology, Feinberg School of Medicine, Northwestern University, Chicago, IL 60611, USA; 2. Center for Reproductive Science, Northwestern University, Evanston, IL 60208, USA; 3. Master of Biotechnology Program, Northwestern University, Evanston, IL 60628, USA; 4. Northwestern Medical Group, Northwestern University, Chicago, IL, 60611, USA; 5. Department of Biomedical Engineering, College of Engineering and Medical School, University of Michigan, Ann Arbor, MI 48109, USA

*Corresponding author: Teresa K. Woodruff, Ph.D., Tel: + 1-321-503-2535; Fax: + 1-312-503-5607; E-mail: tkw@northwestern.edu

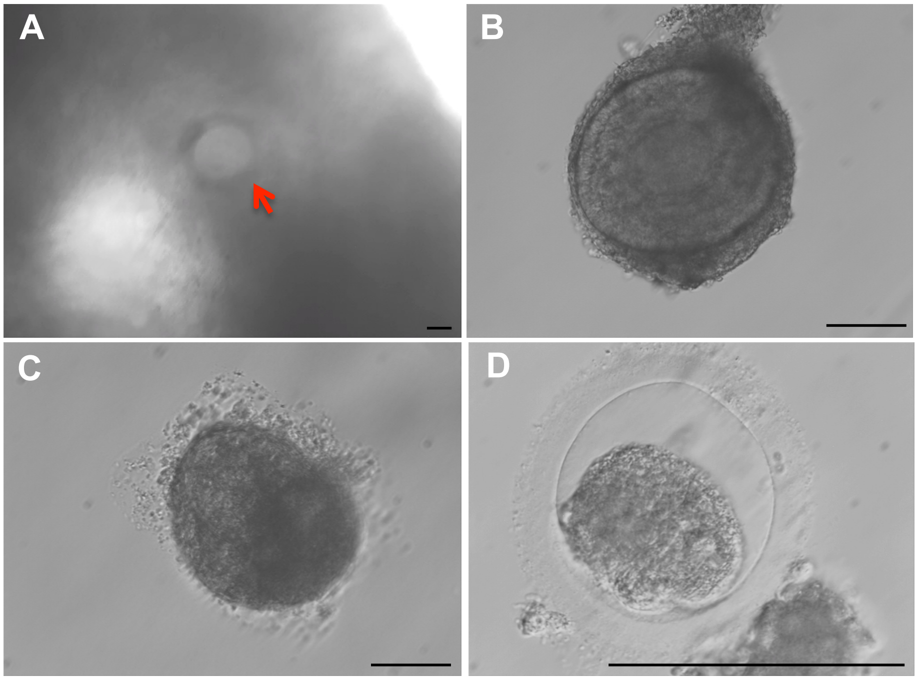

Figure S1. (A-B) Representative images of (A) human ovarian tissue with embedded follicle and (B) a mechanically isolated multilayer secondary follicle. (C-D) Representative images of (A) human follicles cultured on a flat surface and (B) the retrieved oocyte after follicles were cultured for 4-6 days. Red arrow: follicle embedded in the cortex area of ovarian tissue. Scale bar: 100 μm.

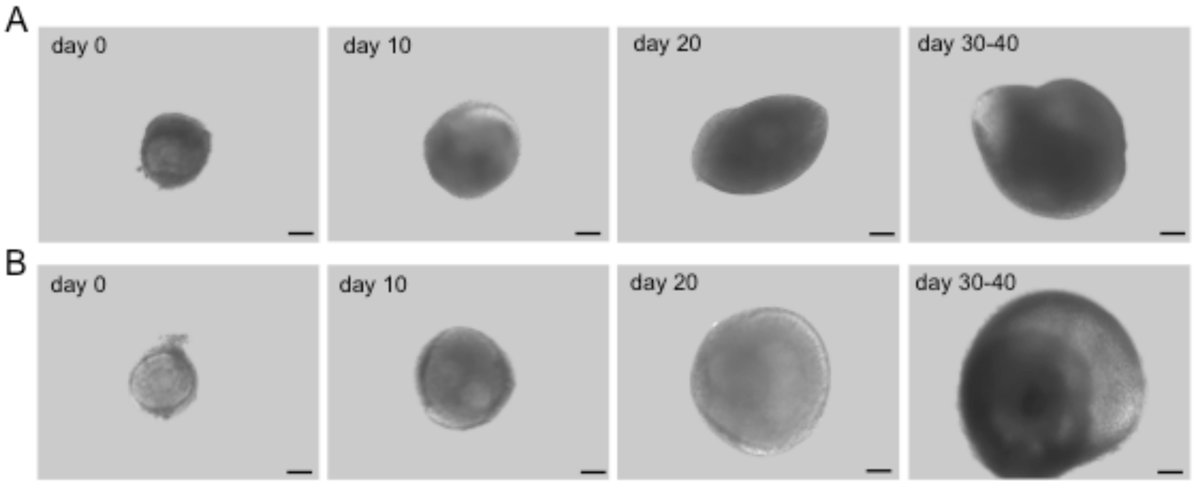

Figure S2. (A) Representative images of follicles cultured only within alginate hydrogels. (B) Representative images of follicles cultured using the two-step strategy. Scale bar: 100 μm.

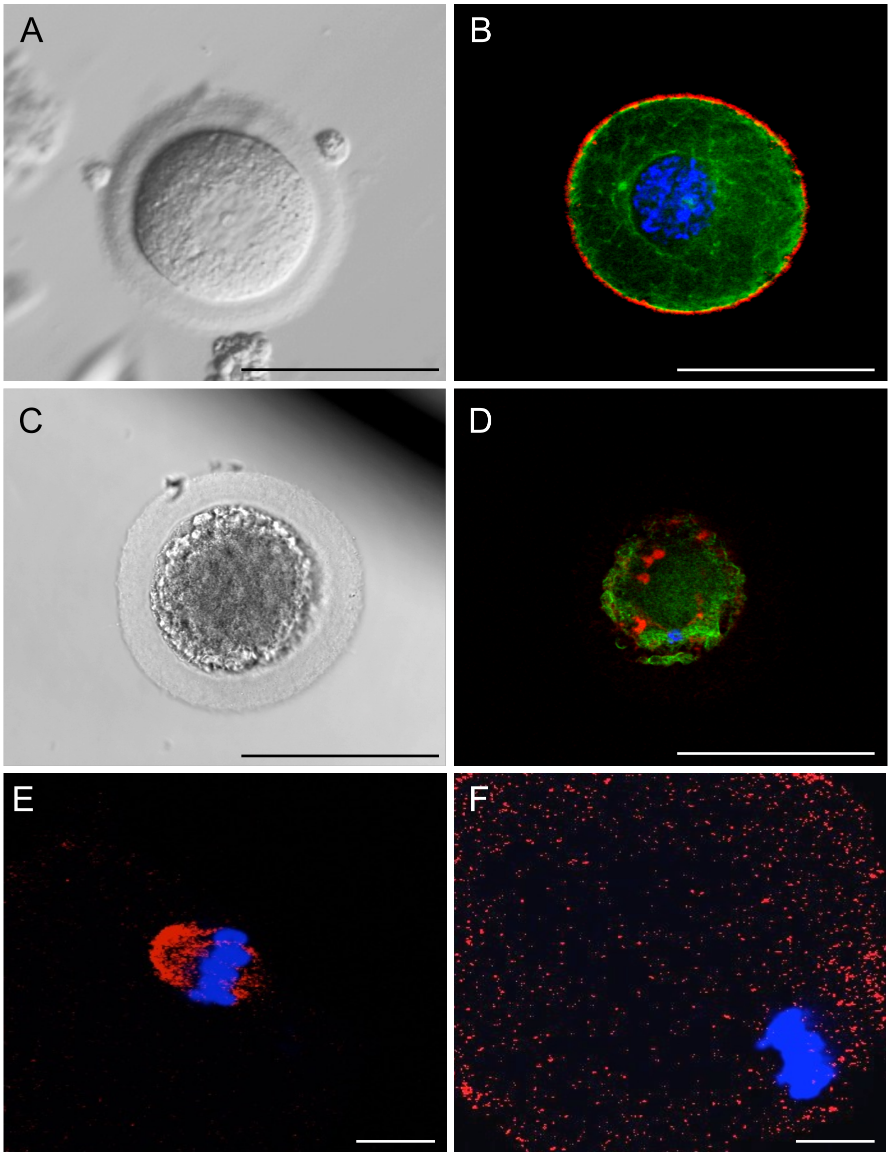

Figure S3. A-D: IVM outcomes of oocytes from follicles cultured only in alginate hydrogel. Oocyte in the GV stage (A-B) and degenerated oocyte (C-D) with immunofluorescence staining of microtubule fibers (green), actin (red), and chromosomes staining (blue). E-F: Representative MII oocytes from follicles cultured using the two-step culture strategy with expression of TPX2 (E) and DAZL (F). Blue staining: chromosomes in E and F; red: TPX2 in E and DAZL in F. Scale bar: 100 μm in A-D and 10 μm in E and F.

Table S1. Characteristics of participants who donated part of their ovarian tissue for research through the National Physician Cooperative (NPC) of the Oncofertility Consortium between January 2014 and February 2015.

Participant	Cancer diagnosis	Age	Previous cancer treatment	Collected multilayer secondary follicles
A	Ovarian mass	31	Yes (Vincristine, dactinomycin, cytoxan, carboplatin and etoposide)	0
B	Acute myeloid leukemia	16	Yes (AAML 1031)	0
C	Hodgkin disease	29	Yes (ABVD chemotherapy)	0
D	Sickle cell disease	6	Yes (Alemtuzumab)	0
E	Cervical cancer	33	Yes (Cerclage)	2
F	High-risk neuroblastoma	11	Yes (children's oncology group pilot trial ANBL09P1)	0
G	Acute myeloid leukemia	12	Yes (Cytarabin)	0
H	Acute myeloid leukemia	19	Yes (cytarabine, etoposide, doxorubicin and mitoxantrone)	3
I	Relapsed Hodgkin disease	18	Yes (Doxorubicin, bleomycin, vincristine, etoposide, prednisone)	0
J	Relapsed Wilm’s tumor	3	Yes (Doxorubicin, bleomycin, vincristine, etoposide, prednisone)	0
K	Hemophagocytic lymphohistiocytosis	0.5	Yes (Etoposide)	0
L	Endometrial stromal sarcoma	30	Yes (Ifosfamide and cyclophosphamide)	0
M	Synovial sarcoma (abdomen)	10	Yes (Ifosfamide and doxorubicin)	0
N	Rhabdomyosarcoma	4	Yes (irinotecan and vincristine)	0
O	Primary mediastinal B cell lymphoma localized to chest	12	Yes (R-DA-EPOCH rituximab)	0
P	Optic pathway pilocytic astrocytoma	14	Yes (vinblastine, selumetinib, and PBTC-029)	0
Q	Embryonal rhabdomyosarcoma	14	Yes (Vincristine and cyclophosphamide)	0
R	Relapsed All	15	Yes (Vincristine and prednisone)	0
S	Acute myeloid leukemia	10	Yes (Vincristine, dexamethasone, doxorubicin (75 mg/m^2), peg-asparaginase, cyclophosphamide)	0
T	NK cell lymphoma	14	Yes, (Ifosfamide and	0

			doxorubicin)	
U	Hodgkin disease	15	Yes, (Pediatric Oncology Group with ABVE-PC)	0
V	High-risk neuroblastoma	4	Yes(cyclophosphamide and topotecan)	0
W	Ewing sarcoma	23	None	0
X	Lymphoma	16	None	13
Y	Glioblastoma multiforme	27	None	2
Z	Hodgkin lymphoma	16	None	2
AA	Small cell carcinoma of ovary	13	None	5
BB	Rectal cancer	25	None	2
CC	Rhabdomyosarcoma	25	None	5
DD	Sickle cell disease	13	None	0
EE	Glioblastoma multiforme	22	None	2
FF	Rectal cancer	37	None	0
GG	Aplastic anemia	7	None	0
HH	Breast cancer	29	None	1
II	Beta-thalassemia	7	None	6
JJ	Synovial sarcoma	17	None	4
KK	Ewing sarcoma	13	None	4
LL	Fanconi anemia	5	None	1
MM	Beta-thalassemia	6	None	5
NN	Aplastic anemia	15	None	6
OO	Sickle cell disease	10	None	0
PP	Pelvic mass	8	None	0
QQ	Aplastic anemia	14	None	0
RR	Rhabdomyosarcoma	28	None	2

Table S2. In vitro follicle growth outcomes of follicles cultured with one step alginate encapsulation and two-step culture strategy

	Collected multilayer secondary follicles	Follicles displayed antrum	One step alginate encapsulation	Follicle cultured with two-step strategy	Follicles produced non-meiotic competent oocyte	Follicle produced meiotic competent oocyte
Number	65	32	12	20	16	4
Percentage	/	/	/	100%	80%	20%
